# Immunogenicity and protective efficacy of a co-formulated two-in-one inactivated whole virus particle COVID-19/influenza vaccine

**DOI:** 10.1038/s41598-024-54421-1

**Published:** 2024-02-20

**Authors:** Chimuka Handabile, Marumi Ohno, Toshiki Sekiya, Naoki Nomura, Tomomi Kawakita, Mamiko Kawahara, Masafumi Endo, Tomohiro Nishimura, Minako Okumura, Shinsuke Toba, Michihito Sasaki, Yasuko Orba, Brendon Y. Chua, Louise C. Rowntree, Thi H. O. Nguyen, Masashi Shingai, Akihiko Sato, Hirofumi Sawa, Kazumasa Ogasawara, Katherine Kedzierska, Hiroshi Kida

**Affiliations:** 1 Institute for Vaccine Research and Development (HU-IVReD), Hokkaido University, Sapporo, Japan; 2 Division of Biologics Development, International Institute for Zoonosis Control, Hokkaido University, Sapporo, Japan; 3 One Health Research Center, Hokkaido University, Sapporo, Japan; 4 International Collaboration Unit, International Institute for Zoonosis Control, Hokkaido University, Sapporo, Japan; 5 Department of Microbiology and Immunology, The University of Melbourne at the Peter Doherty Institute for Infection and Immunity, Melbourne, Australia; 6 Division of International Research Promotion, International Institute for Zoonosis Control, Hokkaido University, Sapporo, Japan; 7 Division of Vaccine Immunology, International Institute for Zoonosis Control, Hokkaido University, Sapporo, Japan; 8 KM Biologics Co. Ltd, Kumamoto, Japan; 9 Shionogi Pharmaceutical Research Center, Shionogi & Company, Limited, Toyonaka, Japan; 10 Division of Molecular Pathobiology, International Institute for Zoonosis Control, Hokkaido University, Sapporo, Japan

**Keywords:** Vaccines, Virology

## Abstract

Due to the synchronous circulation of seasonal influenza viruses and severe acute respiratory coronavirus 2 (SARS-CoV-2) which causes coronavirus disease 2019 (COVID-19), there is need for routine vaccination for both COVID-19 and influenza to reduce disease severity. Here, we prepared individual WPVs composed of formalin-inactivated SARS-CoV-2 WK 521 (Ancestral strain; Co WPV) or influenza virus [A/California/07/2009 (X-179A) (H1N1) pdm; Flu WPV] to produce a two-in-one Co/Flu WPV. Serum analysis from vaccinated mice revealed that a single dose of Co/Flu WPV induced antigen-specific neutralizing antibodies against both viruses, similar to those induced by either type of WPV alone. Following infection with either virus, mice vaccinated with Co/Flu WPV showed no weight loss, reduced pneumonia and viral titers in the lung, and lower gene expression of proinflammatory cytokines, as observed with individual WPV-vaccinated. Furthermore, a pentavalent vaccine (Co/qFlu WPV) comprising of Co WPV and quadrivalent influenza vaccine (qFlu WPV) was immunogenic and protected animals from severe COVID-19. These results suggest that a single dose of the two-in-one WPV provides efficient protection against SARS-CoV-2 and influenza virus infections with no evidence of vaccine interference in mice. We propose that concomitant vaccination with the two-in-one WPV can be useful for controlling both diseases.

## Introduction

Prior to emergence of severe acute respiratory coronavirus 2 (SARS-CoV-2) and subsequent declaration of the coronavirus disease 2019 (COVID-19) pandemic, seasonal influenza viruses, including influenza A and B, accounted for the majority of respiratory viral infections. After the outbreak of the COVID-19 pandemic, morbidities and mortalities resulting from seasonal influenza viruses declined steeply^1–3^ owing to non-pharmaceutical interventions for COVID-19 control, such as global travel restrictions, pro-social behavioral changes and good hygiene practices^4–6^. However, with countries lifting strict control measures and increasing international travel, case numbers and fatalities of seasonal influenza have gradually increased^7,8^. Given that the COVID-19 pandemic is still ongoing, both diseases pose a serious public health threat, especially the risk of co-infection, which is associated with exacerbation of clinical outcomes^9–11^.

To date, vaccination is the most effective and economical method for controlling COVID-19 and seasonal influenza. Mass vaccination efforts against both COVID-19 and influenza could overwhelm healthcare systems, particularly in developing countries, and cause vaccination fatigue and further delays would increase the risk of infection with either virus. For instance, vaccination rates against seasonal influenza were significantly lower in post-COVID-19 years^12,13^ and therefore vulnerable populations may be at risk of severe influenza disease unless they have had an immune boosting caused by natural infection with the seasonal influenza viruses. Thus, there is a need for efficient vaccination strategies. Accordingly, the World Health Organization (WHO) now recommends the concomitant administration of licensed COVID-19 and seasonal influenza vaccines^14^.

Concomitant or combined vaccination can be in different forms, including co-formulation and co-administration^15^. In the context of vaccines, the former implies a single vaccine containing antigens from different microbes administered as a single dose, while the latter refers to the inoculation of different vaccine formulations at the same time, usually on different body parts^15,16^. Currently, there is limited evidence regarding the safety and protectivity of co-formulated COVID-19 and seasonal influenza vaccines. The risk of reduced immunogenicity of one or more antigens in the vaccine and the possible exacerbation of side effects needs to be addressed. Recent evidence has shown successful protection against COVID-19 and influenza by a co-formulated mRNA vaccine encoding the SARS-CoV-2 S protein and hemagglutinin (HA) of influenza virus in mice^17^. However, difficulty in handling mRNA vaccines, particularly requirement and maintenance of cold chain transportation, in addition to potential adverse effects^18–20^, makes their use undesirable for annual vaccination.

Classical inactivated whole virus particle vaccines (WPVs) have been used for preventing seasonal influenza^21^ and other infectious diseases^22–25^. In the production of inactivated vaccines, inactivation is a crucial step to ensure the vaccine is safe for use. Commonly used inactivation methods include the use of formaldehyde, beta-propiolactone, solvents/detergents such as ether and triton X-100 and non-traditional methods including gamma and ultraviolet radiation are employed for virus inactivation^26,27^. Because irradiation methods have not been validated for the production of inactivated vaccines for human use, formaldehyde and B-propiolactone remain widely used for inactivated vaccine preparation. Although they cause structural modification, precise optimization of experimental conditions such as duration of inactivation, temperature, buffer, and concentration of inactivating agent could achieve a more functional product^28^.

As with influenza, inactivated WPV for COVID-19 have been developed and licensed for use in humans, with relatively mild adverse effects compared to mRNA or vectored vaccines^29^. Improved production technologies have alleviated WPV-associated side effects historically linked to contaminants during vaccine preparation. Their potent immunogenicity, ease of handling, stability, long shelf life, and good safety profile render WPVs ideal for annual COVID-19 and influenza vaccination. Furthermore, a study by Bao et al.^10^ showed successful protection from COVID-19 and seasonal influenza in an animal model after vaccination with a co-formulated vaccine containing adjuvanted COVID-19 WPV and influenza split virus vaccine (SV). Another group reported the safety and immunogenicity of the same adjuvanted COVID-19 vaccine co-administered with a seasonal quadrivalent influenza SV in adults^10,30^. However, the use of influenza SV in the Bao et al*.* study may pose a challenge since SV has modest immunogenicity in high-risk groups thus an alternative such as WPV would be appropriate. A two-in-one vaccine utilizing WPV instead of SV may hold a more comprehensive immunization strategy because of the ability to induce broad and balanced immune response, robust induction of antibodies and T cells to a wide array of viral antigens, generation of immunological memory, and good priming potency in naïve individuals^31,32^.

Here, the development of a two-in-one COVID-19/influenza WPV containing antigens of SARS-CoV-2 and seasonal influenza virus strains derived from cell culture and embryonated chicken eggs, respectively, is reported for induction of humoral immunity and protection against SARS-CoV-2 and seasonal influenza viruses. It is shown that a single inoculation with the unadjuvanted WPV elicits an antigen-specific immune response and protects BALB/c mice from SARS-CoV-2 or influenza virus infection.

## Results

### Co-formulation does not interfere with inducing vaccine-specific antibody responses

**Figure 1:**
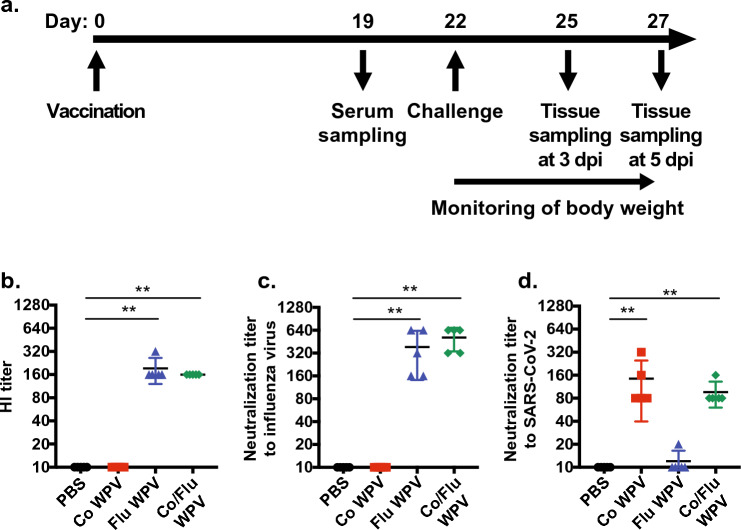
Antibodies against influenza virus and SARS-CoV-2. Female BALB/c mice were immunized subcutaneously with Co WPV, Flu WPV, Co/Flu WPV, or PBS as the control group, and sample collection and virus challenge experiments were conducted (n = 5/group; (**a**). Sera were collected at 19 days post-vaccination for measurement of hemagglutination inhibition (HI) antibody titers (**b**) and neutralizing antibodies against A/California/07/2009 (X-179A)(H1N1) [A/California (H1N1)] (**c**) and SARS-CoV-2 WK-521 (Ancestral strain) (**d**). Mean and SD are shown. Statistical analysis was performed using Kruskal–Wallis test with Dunn’s multiple comparisons tests. **p < 0.01.

We prepared individual WPVs composed of formalin-inactivated SARS-CoV-2 WK-521, (Ancestral strain; Co WPV) or influenza virus A/California/07/2009 (X-179A) (H1N1) pdm, [A/California (H1N1); Flu WPV]. Co WPV and Flu WPV were mixed to produce, a two-in-one vaccine (Co/Flu WPV) containing equal amounts of protein from each vaccine. To assess the induction of virus-specific humoral immunity, we measured neutralizing antibody titers against influenza virus and SARS-CoV-2 using serum obtained from BALB/c mice 19 days after vaccination with a single dose of Co WPV, Flu WPV, Co/Flu WPV, or PBS as the control (Fig. 1a). Mice that received Flu WPV or Co/Flu WPV mounted higher levels of HA inhibiting (HI) and neutralizing antibodies titers against A/California (H1N1) compared to mice inoculated with PBS (Fig. 1b,c). Neutralizing antibody titers against the influenza virus ranged from 160 to 640 in mice vaccinated with Flu WPV or Co/Flu WPV with no statistically significant differences between the groups. As expected, we did not detect HI or neutralizing antibody titers against influenza virus in animals inoculated with either PBS or Co WPV. Neutralizing antibody titers against SARS-CoV-2 were also comparable between animals receiving Co WPV only (range 80–320) and those vaccinated with Co/Flu WPV (range 80–160), which were both higher than PBS control (Fig. 1d). These results reveal that co-formulation of Flu WPV and Co WPV has no apparent interfering effect on antibody production against respective antigens.

### Protection from influenza virus challenge

**Figure 2:**
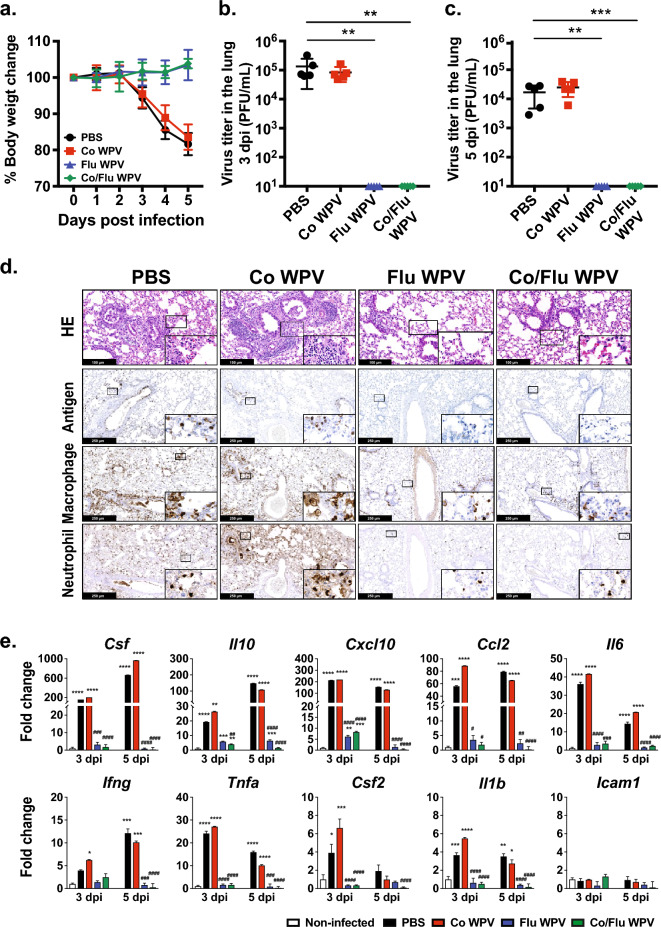
Protection from influenza virus challenge. Female BALB/c mice were immunized subcutaneously with Co WPV, Flu WPV, Co/Flu WPV, or PBS as the control group, and sample collection and virus challenge experiments were conducted as shown in Fig. 1a (n = 10/group). At 22 days post-vaccination, the animals were challenged with 3,000 PFU of A/California (H1N1). Following the influenza virus challenge, body weight change (**a**) and lung viral loads were measured at 3 and 5 dpi (**b** and **c**). Mean and SD are shown. Statistical analysis was performed using Kruskal–Wallis test with Dunn’s multiple comparisons tests. **p < 0.01. Some lung tissues harvested at 5 dpi were used to analyze lung pathology by H&E and immunohistochemistry to detect influenza A virus and macrophage (F4/80) and neutrophil (Ly-6G) markers (**d**). Each lung section is a representative of 5 lung sections analysed. Gene expression levels of pro-inflammatory cytokines and chemokines were measured using real-time RT-PCR (**e**). Gene expression levels are presented as fold changes relative to the non-infected control group. Mean and SD are shown. Statistical analyses were performed on dCt values using two-way ANOVA with Sidak’s multiple comparisons test. The * and # symbols indicate statistical significance between the non-infected group and infected groups and between unvaccinated (PBS) and vaccinated groups, respectively. *p < 0.05, **p < 0.01, ***p < 0.001 ****p < 0.0001 and #*p* < 0.05, ##*p* < 0.01, ###*p* < 0.001 ####*p* < 0.0001.

To investigate whether co-formulated Co/Flu WPV confers protection from influenza virus challenge in vivo*,* BALB/c mice vaccinated with Co WPV, Flu WPV, Co/Flu WPV, or PBS were intranasally challenged with 3000 plaque forming units [3000 PFU ~ 40 × 50% mouse lethal dose 50 (40 MLD50)] of influenza A/California (H1N1) virus on day 22 (Fig. 1a). Body weight of mice in the PBS group and those vaccinated with Co WPV steadily decreased from day 3 onwards by up to 20% of initial body weight before the challenge, while body weight of those vaccinated with Flu WPV and Co/Flu WPV fluctuated within normal ranges (Fig. 2a). To evaluate efficacy of Co/Flu WPV in suppressing virus replication, we measured virus titers in the right lobes of the lungs at 3- and 5-days post-infection (dpi). Virus titers in lung homogenates of the PBS group and Co WPV group were approximately 10^5^ and 10^4^ PFU/mL at 3 and 5 dpi, respectively (Fig. 2b,c). By contrast, virus titers were under the detection limit in lung homogenates from mice vaccinated with Flu WPV or Co/Flu WPV (Fig. 2b,c). These observations were consistent with the results of immunohistochemistry staining for the influenza virus, where viral antigens were not detected in the lungs of mice inoculated with either Flu WPV or Co/Flu WPV at 5 dpi, suggesting efficient protection by the vaccine (Fig. 2d).

We further examined the protective efficacy of Co/Flu WPV and Flu WPV from influenza-associated pathological changes in the lungs on hematoxylin and eosin (H&E) stained lung sections (Fig. 2d). Five days after influenza virus challenge, mice in the PBS and Co WPV groups showed diffuse pneumonia with increased alveolar wall thickness, infiltration of inflammatory cells, denudation of bronchial epithelium, and deposition of necrotic debris in the bronchial lumen. In addition, perivascular and peribronchiolar cuffing was observed. Infiltrating inflammatory cells mainly consisted of macrophages and neutrophils as shown by immunostaining using anti-F4/80 and anti-Ly-6G antibodies. Conversely, mice that received Flu WPV or Co/Flu WPV showed very mild pathology which differed only slightly from lung sections of non-infected control group, suggesting that these animals were protected from influenza virus-induced pneumonia (Fig. 2d and Supplemental figs. S1 and S2a).

To further investigate protection from pulmonary pathology, we measured gene expression of *colony stimulating factor 2* (*Csf2)*, *Csf3, interleukin- 1 beta* (*Il1b*)*, Il6, Il10, interferon-gamma* (*Ifng*)*, tumor necrosis factor* (*Tnf*), *C-X-C motif chemokine ligand 10* (*Cxcl10*), *C–C motif chemokine ligand 2* (*Ccl2*), and *intracellular adhesion molecule 1* (*Icam1*) in lung tissue collected at 3 and 5 dpi (Fig. 2e). After influenza virus challenge, the expression of majority of inflammatory genes was elevated in PBS- and Co WPV-vaccinated groups, but much lower in animals vaccinated with Flu WPV or Co/Flu WPV at both sampling time points, consistent with the histology data. There was no major difference between gene expression levels in mice vaccinated with Flu WPV, Co/Flu WPV or the non-infected control group, although *Csf, Cxcl10* and *Il10* levels in Flu WPV and Co/Flu WPV mice were slightly higher than non-infected control mice. Together, our results suggest that the co-formulated Co/Flu WPV can protect mice from influenza virus challenge.

### Protection from SARS-CoV-2 infection

**Figure 3:**
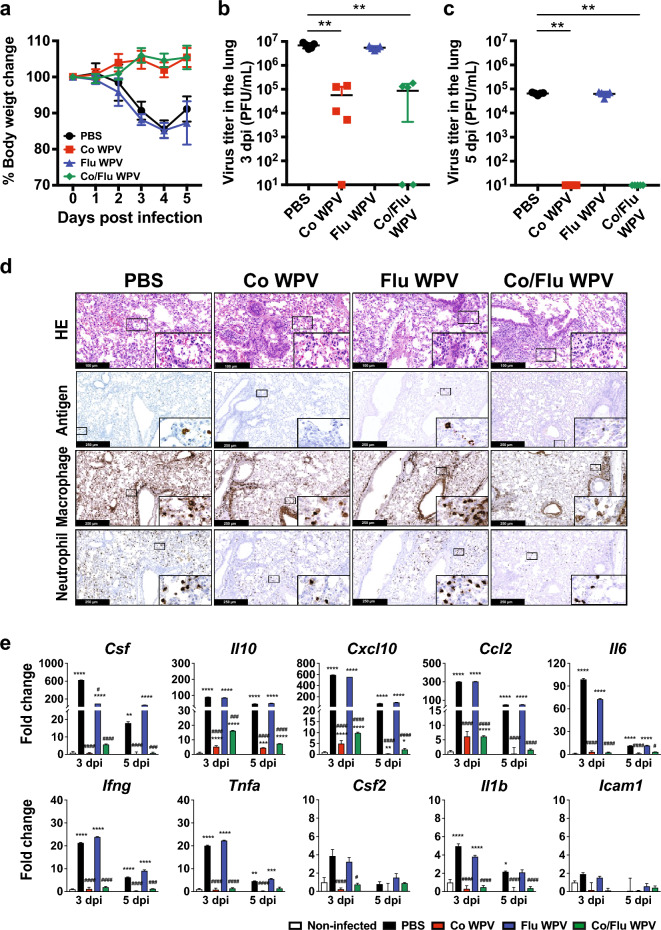
Protection from SARS-CoV-2 challenge. Female BALB/c mice were immunized subcutaneously with Co WPV, Flu WPV, Co/Flu WPV, or PBS as the control group, and sample collection and virus challenge experiments were conducted as shown in Fig. 1a (n = 10/group). At 22 days post-vaccination, the animals were challenged with 10^5^ PFU of mouse-adapted SARS-CoV-2 (SARS-CoV-2 MA-P10). Following virus challenge, body weight change was monitored (**a**) and lung viral loads were measured at 3 and 5 dpi (**b** and **c**). Mean and SD are shown. Statistical analysis was performed using Kruskal–Wallis test with Dunn’s multiple comparisons tests. **p < 0.01. Some lung tissues harvested at 5 dpi were used to analyze lung pathology by H&E and immunohistochemistry to detect SARS-CoV-2 nucleocapsid and macrophage (F4/80) and neutrophil (Ly-6G) markers (**d**). Each lung section is a representative of 5 lung sections analysed. Gene expression levels of pro-inflammatory cytokines and chemokines were measured using real-time RT-PCR (**e**). Gene expression levels are presented as fold changes relative to the non-infected control group. Mean and SD are shown. Statistical analyses were performed on dCt values using two-way ANOVA with Sidak’s multiple comparisons test. The * and # symbols indicate statistical significance between the non-infected group and infected groups and between unvaccinated (PBS) and vaccinated groups, respectively. *p < 0.05, **p < 0.01, ***p < 0.001 ****p < 0.0001 and #*p* < 0.05, ##*p* < 0.01, ###*p* < 0.001 ####*p* < 0.0001.

We then investigated the protectivity of Co/Flu WPV against SARS-CoV-2 infection as we did with the influenza challenge model above. Vaccinated BALB/c mice were challenged with 10^5^ PFU of mouse-adapted SARS-CoV-2 Ancestral strain (SARS-CoV-2 MA-P10^33^), 22 days after vaccination. At 4 dpi, animals vaccinated with Flu WPV or PBS lost up to 15% of their initial body weight while those vaccinated with Co WPV or Co/Flu WPV did not lose body weight throughout the period of observation (Fig. 3a). While infectious virus was detected in the lungs of all animals at 3 dpi, animals vaccinated with Co WPV or Co/Flu WPV had approximately 100-fold lower virus titers than those in the PBS and Flu WPV groups (Fig. 3b). Moreover, some mice vaccinated with Co WPV or Co/Flu WPV did not have detectable virus at 3 dpi indicating protection by vaccine-induced immunity, and by 5 dpi, these two groups had no culturable virus detected in the lung homogenates (Fig. 3b,c). Consistently, immunostaining analyses revealed lower signals of SARS-CoV-2 nucleocapsid in the lungs of animals vaccinated with Flu WPV or PBS, but not in those vaccinated with Co WPV or Co/Flu WPV (Fig. 3d). However, histopathological analyses with H&E showed clear pneumonia as indicated by increased infiltration of leukocytes in the peri-bronchiolar and alveolar areas, thickening of alveolar septa, perivascular cuffing, and deposition of necrotic debris in the bronchial lumen in the lungs of all immunized and non-immunized mice at 5 dpi (Fig. 3d and Supplemental Fig. S2b). Infiltrating inflammatory cells were mainly characterized by macrophages and neutrophils as seen in immunostained lung sections using anti-F4/80 and anti-Ly-6G antibodies.

Contrary to the histopathological findings, the induction of pro-inflammatory cytokines at 3 and 5 dpi was suppressed by Co WPV and Co/Flu WPV (Fig. 3e). Many of the cytokines and chemokines that have been implicated in severe COVID-19 infection, including *Il6, Tnf,* and *Ilb*^34^ were increased in the PBS- and Flu WPV-inoculated groups but not in mice vaccinated with Co WPV or Co/Flu WPV. Gene expression levels of cytokines and chemokines in PBS and Flu WPV groups were highest at 3 dpi and reduced at 5 dpi, which showed similar kinetics with the lung viral titers. Conversely, gene expression levels in the animals vaccinated with Co WPV or Co/Flu WPV, either showed no difference with the non-infected control group or were mildly induced at 3 dpi, such as *Il10*, *Cxcl10*, and *Ccl2,* but these were much lower than PBS and Flu WPV groups. Collectively, these results demonstrated the protectivity of Co/Flu WPV against SARS-CoV-2 infection, which is comparable to that of Co WPV, in aspects of suppressing viral replication and cytokine/chemokine responses in the lung and halting weight loss. However, the beneficial effect of the vaccines on improving pneumonia was not clear. This point was further investigated in the next section.

### Practical two-in-one vaccine with quadrivalent seasonal influenza vaccine

**Figure 4:**
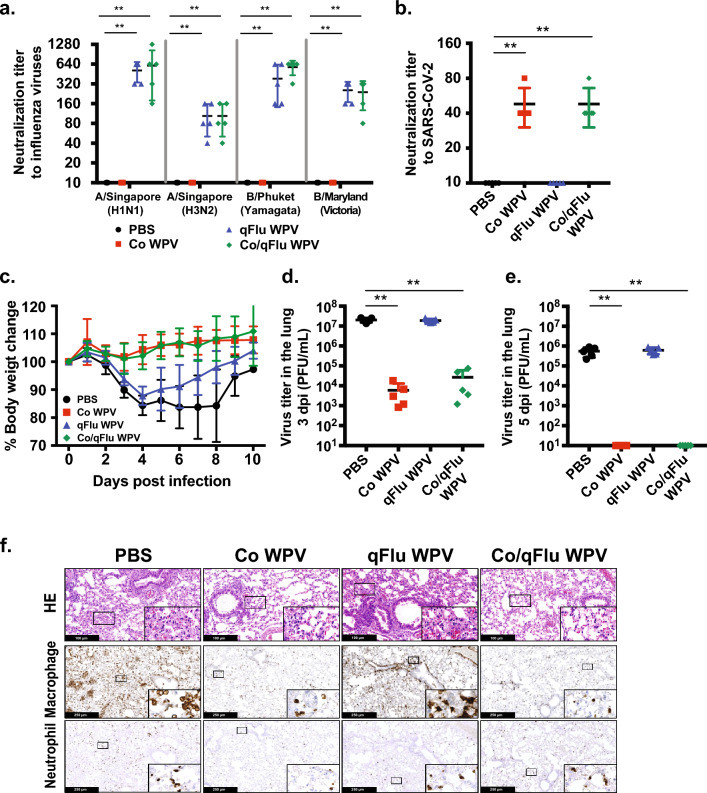
Practical two-in-one vaccine with quadrivalent seasonal influenza vaccine. Female BALB/c mice were immunized subcutaneously with Co WPV, qFlu WPV, Co/qFlu WPV, and PBS as the control group (n = 13/group). Sera were collected at 19 days post-vaccination for measurement of neutralizing antibodies against A/Singapore/GP1908/2015 (IVR-180) (H1N1), A/Singapore/INFIMH-16–0019/2016 (IVR-186) (H3N2), B/Phuket/3073/2013 (Yamagata lineage) and, B/Maryland/15/2016 (NYMC BX-69A) (Victoria lineage) (a), and SARS-CoV-2 (**b**). Mean and SD are shown. Statistical analysis was performed using Kruskal–Wallis test with Dunn’s multiple comparisons tests. **p < 0.01(**a**), **p < 0.01 (**b**). At 22 days post-vaccination, the animals were challenged with 10^5^ PFU of SARS-CoV-2 MA-P10. Following virus challenge, body weight change was monitored (c) and lung viral loads were measured at 3 and 5 dpi (**d** and **e**). Mean and SD are shown. Statistical analysis was performed using Kruskal–Wallis test with Dunn’s multiple comparisons tests. **p < 0.01. Some lung tissues were harvested at 10 dpi for lung pathology and immunohistochemistry (**f**). For histopathological analysis, lung sections were stained with H&E, and immunohistochemistry was performed to detect SARS-CoV-2 nucleocapsid and macrophage (F4/80) and neutrophil (Ly-G6) markers. Each lung section is a representative of 3 lung sections analysed.

Since currently licensed influenza vaccines are available as trivalent or quadrivalent formulations, we prepared Co/qFlu WPV by co-formulating Co WPV with quadrivalent influenza WPV (qFlu WPV) containing antigens for the 2018–2019 flu season; ﻿A/Singapore/GP1908/2015 (IVR-180) (H1N1) pdm09 [A/Singapore (H1N1)], A/Singapore/INFIMH-16-0019/2016 (IVR-186) (H3N2) [A/Singapore (H3N2)], B/Phuket/3073/2013 (Yamagata lineage) [B/Phuket (Yamagata)] and B/Maryland/15/2016 (NYMC BX-69A) (Victoria lineage) [B/Maryland (Victoria)]. To evaluate its immunogenicity and protective efficacy, BALB/c mice were vaccinated with Co WPV, qFlu WPV, Co/qFlu WPV, or PBS as the control group, and neutralization antibody titers in the collected serum were compared. Neutralizing antibody titers in the serum against all respective influenza virus strains were successfully induced on day 22 from vaccination and comparable (range 40–1280) between animals vaccinated with the qFlu WPV alone or Co/qFlu WPV (Fig. 4a). We also observed induction of neutralizing antibody titers in the serum against SARS-CoV-2 in animals vaccinated with Co WPV and Co/qFlu WPV (range 40–80) while neutralizing antibody titers in control animals were below the detection limit (Fig. 4b). These results show that co-formulated Co/qFlu WPV induces humoral immune responses to their respective viruses without apparent interference with each other.

We subsequently investigated the protectivity of Co/qFlu WPV against SARS-CoV-2 infection challenge, in which the experimental duration was extended up to 10 days to observe the vaccine effect on disease severity at the later phase. Following inoculation of 10^5^ PFU of SARS-CoV-2 MA-P10 at day 22 post-vaccination, mice vaccinated with Co WPV or Co/qFlu WPV showed no weight loss, while all control animals and mice vaccinated with qFlu WPV showed steep weight loss until 4 dpi and then slowly recovered, although qFlu WPV-vaccinated animals begun recovery of body weight earlier than the PBS group (Fig. 4c). Lung virus titers of animals in the PBS and qFlu WPV groups had on average 2 × 10^7^ and 1.9 × 10^7^ PFU at 3 dpi, and 5.5 × 10^5^ and 6.2 × 10^5^ PFU at 5 dpi, respectively (Fig. 4d,e). In contrast, mice vaccinated with Co WPV or Co/qFlu WPV had lower viral loads after virus challenge at 3 and 5 dpi. Consistently, immunostaining analyses revealed lower signals of SARS-CoV-2 nucleocapsid in the lungs of animals vaccinated with Co WPV or Co/qFlu WPV, compared to animals vaccinated with PBS or qFlu WPV at 3 and 5 dpi (Supplemental Fig. S4a and b). Of note, the protectivity against SARS-CoV-2 MA-P10 exhibited by Co/Flu WPV and Co/qFlu WPV was similar, suggesting co-formulation of Co WPV with monovalent or quadrivalent vaccines may not affect protectivity from COVID-19.

As the next step, we examined the effect of vaccines on infection-induced lung pathology at early to later stages by analyzing H&E-stained lung sections collected at 3, 5, and 10 dpi. At 3 and 5 dpi, the lungs showed massive accumulation of immune effector cells, thickening of the alveolar wall, sloughing of the bronchial epithelium, deposition of necrotic debris, and peri-vascular cuffing in all mice regardless of vaccination status (Supplemental Figs. S3a and b), which was consistent with the histopathological findings in the first SARS-CoV-2 challenge experiment described with monovalent vaccines (Fig. 3d). Moreover, there was no difference in histology scores of the infected lungs among groups at 3 and 5 dpi (Supplemental Fig. S4a). We also assessed the frequency of macrophages and neutrophils in lung sections by immunostaining using anti-F4/80 and anti-Ly-6G antibodies, respectively. Relative to the non-infected control animals, all infected animals had increased numbers of macrophages (F4/80^+^ cells) at 3 dpi with the highest numbers in Co/qFlu-vaccinated animals (1057 ± 198.3; Supplemental Fig. S3a and S4b) At 5 dpi, the macrophages increased further in all vaccinated animals except for those vaccinated with the Co/qFlu WPV which showed a slight decrease compared to 3 dpi (means of 1057 at 3 dpi vs 864.6 at 5 dpi; Supplemental Fig. S3b and S4b). On the other hand, the frequency of neutrophils (Ly-6G^+^ cells) was higher in PBS control and qFlu WPV-vaccinated animals than in Co WPV- or Co/qFlu-vaccinated animals at 3 and 5 dpi (Supplemental Fig.S3 and 4c). Therefore, from the findings of histopathology and immunostaining, it was suggested that the protective effect of vaccination with Co WPV regardless of co-formulation with influenza monovalent or quadrivalent WPVs on lung pathology may not be apparent during the early stage of SARS-CoV-2 infection.

On the other hand, the effect of Co WPV or Co/qFlu WPV on the lung histological changes was clearly observed at 10 dpi, the later phase of the disease (Fig. 4f and Supplemental Fig. S4a). PBS control animals and those vaccinated with qFlu WPV showed severe lung inflammation than at early time points, even when body weight was on a way to recovery, as indicated by thickened alveolar walls, marked deposition of debris in alveolar spaces, vascular damage, and higher frequency of macrophages than at early time points. While the difference in the numbers of macrophages at 10 dpi between groups did not reach significance, those in the Co WPV and Co/qFlu WPV groups had approximately half the number recorded at 5 dpi while the numbers in the PBS and qFlu WPV were similar to those recorded at 5 dpi (Fig. 4f and Supplemental Fig. S4b). Ly-6G^+^ cells in all vaccinated animals however returned to baseline levels by 10 dpi as in the non-infected control animals (Supplemental Fig. S4c). In contrast, lung tissues collected from animals vaccinated with Co WPV or Co/qFlu WPV showed much less inflammation than control animals (Fig. 4f and Supplemental Fig. S4a). Altogether, vaccination with Co/Flu and Co/qFlu WPV significantly reduces the severity of SARS-CoV-2 infection in mice and ameliorates SARS-CoV-2-induced lung damage.

### Induction of cross-reactive neutralizing antibodies

**Figure 5:**
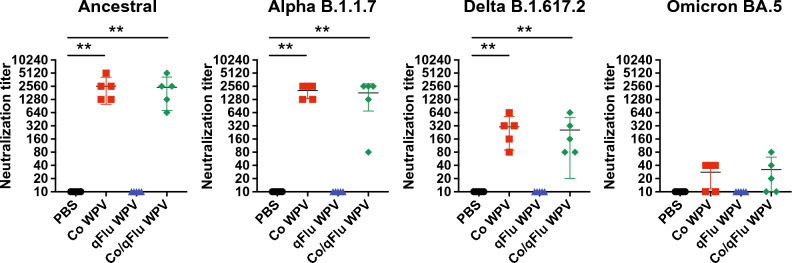
Cross-neutralizing antibodies against SARS-CoV-2 variants. Female BALB/c mice were immunized twice subcutaneously with Co WPV, qFlu WPV, Co/qFlu WPV, or PBS as the control group at day 0 and day 19 (n = 5/group). Sera were collected 2 weeks after the second vaccination (36 days after initial vaccination) for measurement of neutralizing antibodies against SARS-CoV-2 Ancestral strain and Alpha (B.1.1.7), Delta (B.1.617.2), and Omicron (BA.5) variants. Mean and SD are shown. Statistical analysis was performed using Kruskal–Wallis test with Dunn’s multiple comparisons tests. **p < 0.01.

Under selection pressure of host immunity induced by infections or vaccinations with previous prevalent strains, novel strains that could evade established immunity have been continuously emerging: several strains had been defined as variants of concern (VOCs) by the WHO. Emergence of VOCs has been a major challenge in controlling the COVID-19 pandemic. We sought to determine whether our vaccine formulations mount effective antibody responses against some SARS-CoV-2 VOCs following a two-dose regimen. For this purpose, BALB/c mice were vaccinated with Co WPV, qFlu WPV, or Co/qFlu WPV at day 0 and day 22. Serum samples were collected 14 days after the second vaccination to evaluate neutralizing antibodies against the Ancestral, Alpha (B.1.1.7), Delta (B.1.6.17.2), and Omicron (BA.5) strains (Fig. 5). After two doses, very high neutralizing antibodies were generated against the Ancestral and Alpha variant in animals vaccinated with Co WPV or Co/qFlu WPV reaching mean values of 2560 and 2463 against the Ancestral strain and 2048 and 1808 against the Alpha variant, respectively. We also detected reasonable levels of neutralizing antibody titers against the Delta variant with means of 304 and 256 in animals vaccinated with Co WPV or Co/qFlu WPV, respectively. In contrast, neutralizing antibody titers against the Omicron variant were very low (means of 28 and 32 for Co WPV and Co/qFlu WPV, respectively), and in some animals, the levels were below the detection limit, aligning with human data that a two-dose vaccine regimen may not be sufficient to mount robust humoral immunity against the Omicron variants. Not surprisingly, neutralizing antibody titers in animals inoculated with PBS or qFlu WPV were below the detection limit against all viruses tested. Nonetheless, these results suggest that Co/qFlu WPV can induce cross-reactive neutralizing antibody titers.

## Discussion

Since the COVID-19 pandemic, mRNA vaccines have been used primarily in developed countries such as the United States and Europe. Like seasonal influenza, COVID-19 has spread globally, and annual vaccination is discussed. Given the ease of mRNA vaccine production, such as in vitro synthetic capability and the fact that they can be produced once the genetic information is known, mRNA vaccines are very useful vaccines in the early phase of pandemics of novel viruses that have yet to be well characterized. However, mRNA vaccines may not be suitable for use as annual vaccines because of their strong adverse reactions as well as cold chain problems^35–37^. Therefore, there is a need for safe, easy-handling, and effective vaccine options other than mRNA vaccines. In this study, we report the development of a two-in-one vaccine against COVID-19 and seasonal influenza in terms of immunogenicity and protective efficacy in BALB/c mice.

Although several inactivated COVID-19 vaccines have been developed and demonstrated immunogenicity and protectivity in animals and humans^38–41^, there are scant data demonstrating protection after a single vaccination and without the use of adjuvants. In our study, we showed that a single inoculation of unadjuvanted COVID-19 WPVs significantly prevented body weight loss, gene induction of pro-inflammatory molecules, and reduced virus titers in the lungs following SARS-CoV-2 infection, suggesting that our vaccine is sufficiently immunogenic and protective in mice, comparable to other studies using approved inactivated COVID-19 vaccines. The immunogenicity and protectivity of Flu WPV were comparable to those of the seasonal influenza vaccines used in our previous studies^32,42,43^, thereby further validating our vaccine design, product quality, and reliability. Furthermore, given comparable antibody titers in mice vaccinated with co-formulated WPV and individual WPVs against both viruses, co-formulation of either monovalent or quadrivalent Flu WPV with Co WPV does not seem to interfere with each other in inducing antibodies against SARS-CoV-2 and seasonal influenza viruses. It is consistent with several studies in humans reporting the concomitant administration of licensed inactivated trivalent or quadrivalent seasonal influenza vaccines with currently used COVID-19 vaccines^30,44–47^. Co-administration of either BNT162b2^44^, mRNA-1273 ^47^, ChAdOx1 nCoV-19^44^, BBIBP-CorV^45^, with quadrivalent seasonal influenza viruses reported no safety concerns or interference in immunogenicity except for the NVX-CoV2373^46^ and CoronaVac^30^. Regarding these vaccines, it has been reported that a mild reduction in anti- spike protein IgG was demonstrated after administering NVX-CoV2373 with quadrivalent influenza vaccine particularly after the first dose while in the CoronaVac study a mild reduction in antibodies to SARS-CoV-2 was observed mainly after the second dose of CoronaVac co-administration with quadrivalent influenza vaccine. Yet, in both studies the vaccine immunogenicity in terms of antibody induction using a co-formulation regimen was still considered sufficient, and the clinical relevance of the moderate reductions remain to be investigated. In all the above studies, COVID-19 and influenza vaccines were administered separately in contralateral arms on the same day or on different dates. On the other hand, our study directly demonstrated a possibility that COVID-19 and influenza vaccines can be co-formulated and simultaneously administered to induce robust immune responses. A vaccination strategy utilizing a single vaccine with influenza virus and SARS-CoV-2 antigens, as in our study, may be more advantageous in terms of improving the timely delivery of vaccines and minimizing stress on the healthcare system, particularly during mass campaigns or in developing or remote communities while maintaining the immunogenicity of individual vaccines.

It is important to note that unadjuvanted 9 µg total protein of each virus antigen was administered in this study which is much higher than other reported COVID-19 inactivated vaccines^10,38–41^. While we have previously shown the efficacy of influenza WPV at lower doses^29^, further studies are needed to investigate the efficacy of the COVID-19 WPV at doses lower than 9 µg. In addition, considering that our vaccine is a whole virus, the ability to induce antibodies against other viral proteins such as the N protein, which have been shown to contribute to improved control of SARS-CoV-2, needs to be investigated^48,49^.

Although influenza-induced pneumonia was effectively blocked by Flu WPV and Co/Flu WPV in the challenge experiment of this study, the beneficial effect of either Co WPV, Co/Flu WPV, or Co/qFlu WPV on pathological changes after SARS-CoV-2 infection was not observed at early time points (3 and 5 dpi). The higher virus PFU used in the SARS-CoV-2 challenge experiment compared to that used in the influenza virus challenge (10^5^ PFU vs 3000 PFU) coupled with lower neutralizing antibody titers against SARS-CoV-2 compared to those against influenza viruses (40–160 for SARS-CoV-2 vs 320–640 for influenza virus on average) may partly explain the differences in lung pathology. However, subsequent experiments demonstrated the beneficial effects of our vaccines at the later stage (10 dpi) of SARS-CoV-2 infection. At this stage, mice in the PBS control and Flu WPV groups showed severe signs of pneumonia with massive infiltration of macrophages although the reduced weight had restored to over 95% of the original weight and animals had undetectable viral antigens in the lungs, as observed in the COVID-19 hamster model at 9 dpi^50^. In contrast, mice vaccinated with Co WPV or Co/q WPV showed lesser tissue damage and a low frequency of macrophages in the lung similar to those in uninfected mice, which suggests the earlier resolution of inflammatory responses. It is considered that the efficient reduction of virus replication at early time points, owing to the presence of protective immunity in animals vaccinated with Co WPV and Co/qFlu, attenuated subsequent inflammatory responses and production of chemokines and cytokines, thereby inhibiting aggressive and prolonged pneumonia.

Immune cross-reactivity is one of the key features required for successful COVID-19 vaccines. Two doses of the two-in-one Co/qFlu WPV based on the Ancestral strain were demonstrated to highly induce antibodies against the Alpha strain followed by the Delta strain in this study, as with previous studies investigating combined COVID-19 and influenza vaccines in mouse models using platforms of lipid nano particle-encapsulated mRNA vaccine^17,51^ and adenovirus-based vaccine containing expression vector for SARS-CoV-2 receptor binding domain fused to the stalk of influenza HA^17,51^. Currently, the most dominant strain circulating around the world is the Omicron variant which has the highest number of amino acid substitutions, deletions, and insertions in the spike protein leading to reduced neutralization by antibodies reactive to the Ancestral strain^52^. Indeed it is reported that two doses of the licensed COVID-19 vaccines are not sufficient to neutralize this variant^53,54^. This is similar to our study's result that the co-formulated Co/qFlu WPV induced very low–undetectable antibodies against the Omicron. Data on the induction of cross-reactive antibodies against Omicron by co-formulated/co-administered COVID-19 and influenza vaccines are not yet reported, however, previous studies have revealed that neutralizing capacity of antibodies induced by approved COVID-19 vaccines administered alone can be improved by three-dose vaccination regimens in naïve individuals or two-dose-regimen in COVID-19 convalescents^55–57^. Therefore, annual vaccination with COVID-19 vaccines comprising predominating variants may become necessary, recapitulating the need for co-formulated COVID-19 and seasonal influenza vaccines. In conclusion, our findings present a significant step in the development of a dual purpose COVID-19 and influenza vaccine. Further research, pre-clinical and clinical trials are needed to refine and validate the two-in-one vaccine bringing us closer to an efficient strategy to combat these respiratory infections.

## Materials and methods

### Cells and viruses

RPMI 1640 (Thermo Fisher Scientific, MA, USA) and Dulbecco’s Modified Eagle Medium (DMEM; Thermo Fisher Scientific) were used to grow Mardine-Darby canine kidney (MDCK) cells and transmembrane serine protease 2 (TMPRSS2)-expressing Vero E6 (Vero TMPRSS2) cells, respectively. Both media were supplemented with 10% heat-inactivated fetal calf serum (FCS; GE Healthcare UK Ltd, Little Chalfont, Buckinghamshire, UK), 1 mM of sodium pyruvate (Thermo Fisher Scientific), 50 μM of 2-mercaptoethanol (Merck, Darmstadt, Germany), 100 U/mL of penicillin (Thermo Fisher Scientific), 100 μg/mL of streptomycin (Thermo Fisher Scientific), and 20 μg/mL of gentamicin (Thermo Fisher Scientific). These cells were used for the neutralization and plaque assays. Influenza viruses A/California (H1N1), ﻿A/Singapore (H1N1), A/Singapore (H3N2), B/Phuket　(Yamagata), and B/Maryland (Victoria), SARS-CoV-2 WK-521 (Ancestral strain), Alpha (B.1.1.7, QHN002), Delta (B.1.617.2, TY11-927), and Omicron (BA.5, TY41-702) variants were kindly provided by the National Institute of Infectious Diseases (NIID) in Japan. The mouse-adapted SARS-CoV-2 MA-P10 was kindly provided by Dr. Sato (Shionogi & Co., Ltd, Osaka, Japan) and Dr. Sawa (Hokkaido University). Influenza viruses were propagated in 10-day embryonated chicken eggs while SARS-CoV-2 was propagated in Vero TMPRRS2 cells. Following the propagation of influenza viruses in embryonated eggs, the allantoic fluids were harvested and stored at –80 °C until use. SARS-CoV-2 was propagated in Vero TMPRSS2 cells and the culture supernatant was harvested and then stored at − 80 °C until use.

### Vaccines

Co WPV was prepared from the SARS-CoV-2 Ancestral strain. The virus was propagated in Vero TMPRRS2 and after 2 days of culture at 37 °C, 5% CO_2_, centrifugation was performed at 800 *g*, 4 °C for 30 min for debris clarification. Then 10% formaldehyde was added to the supernatant (final concentration of 0.1%) and incubated for one week at 4 °C for virus inactivation. Following inactivation, the virus was concentrated by centrifugation at 53,900 *g*, 4 °C for 2 h then purified by sucrose density ultracentrifugation at 107,000 *g*, 4 °C for 1.5 h. The total protein concentration was measured using ﻿Nanodrop One spectrophotometer (Thermo Fisher Scientific). Flu WPV was prepared from A/California (H1N1) as previously described^43^. qFlu WPV containing the 2018–2019 virus strains [A/Singapore (H1N1), A/Singapore (H3N2), B/Phuket (Yamagata), B/Maryland (Victoria)] was kindly provided by KM biologics Co. Ltd (Kumamoto, Japan). Co WPV was mixed with Flu WPV or qFlu WPV to produce a two-in-one vaccine (Co/Flu WPV or Co/qFlu WPV) containing equal amounts of protein from each vaccine.

### Animals

Six-week-old female BALB/c mice were purchased from Hokudo, Co., Ltd. (Sapporo, Japan), and housed in BSL-2 and BSL-3 animal rooms at the International Institute for Zoonosis Control under standard laboratory conditions (temperature of 22 °C ± 2 °C, humidity of 50% ± 10%, and a 12 h/12 h light/dark cycle). All mice were given food pellets (CE-2; CLEA Japan, Sapporo, Japan) and water ad libitum. All mice experiments were performed under the approval (approval number:20–0070) of the Animal Care and Use Committee of Hokkaido University following Fundamental Guidelines for Proper Conduct of Animal Experiment and Related Activities in Academic Research Institutions under the jurisdiction of the Ministry of Education, Culture, Sports, Science and Technology in Japan. This study was carried out in compliance with the ARRIVE guidelines.

### Vaccination and serum collection

Seven-week-old BALB/c mice were inoculated once or twice subcutaneously with PBS as a control or either WPV containing 9 µg protein of each viral antigen; Co WPV, Flu WPV, qFlu WPV, Co/Flu WPV, or Co/qFlu WPV. Sera were collected at 19 days (for one dose) or 36 days (for two doses) post-first vaccination from the caudal vena cava after euthanasia for measuring neutralizing antibody titers.

### Virus neutralization assay

The neutralization assay was performed to measure neutralizing antibody titers in the serum of vaccinated mice as previously described for influenza viruses^42,43^. To measure neutralizing antibody titers against SARS-CoV-2, monolayers of Vero TMPRSS2 cells were prepared in 6-well plates by seeding 1.425 × 10^6^ cells/well in 3 mL DMEM and incubated overnight at 37 °C in 5% CO_2_. Mouse sera were inactivated at 56 °C for 30 min and serially diluted two-fold in 96-well microplates to which 100 PFU of SARS-CoV-2 was added and incubated for 1 h at room temperature. Following washing of Vero TMPRSS2 cells with DMEM_anti_, (containing ﻿100 U/mL of penicillin, 100 μg/mL of streptomycin, and 20 μg/mL of gentamicin) the virus-serum mixture was transferred to 6-well plates containing the Vero TMPRSS2 cells and incubated at 37 °C in 5% CO_2_, with shaking every 15 min. Finally, ﻿warmed L15 overlay medium [consisting of Leibovitz L-15 (Life Technologies Corp., Carlsbad, NY, USA) with glutamine supplemented with 100 U/mL penicillin, 100 μg/mL streptomycin, 0.028% (w/v) NaHCO_3_ and 0.9% (w/v) agarose] was added to the 6-well plates. The plates were incubated for three days at 37 °C in 5% CO_2_ and the neutralizing antibody titer was determined as the reciprocal of the highest dilution that prevented the growth of plaques to 50% of that obtained in the control wells.

### Virus challenge and measurement of viral loads

Vaccinated mice were inoculated with either 3000 PFU of A/California (H1N1) [3000 PFU ~ 40 × 50% mouse lethal dose 50 (40 MLD50)] or 10^5^ PFU of SARS-CoV-2 MA-P10^33^ (sublethal dose) intranasally 22 days after vaccination and monitored daily for body weight changes. At 3 and 5 dpi, some mice were humanely euthanized, and lung tissues (right lobes) were harvested for measurement of virus titers and gene expression of proinflammatory cytokines and chemokines. Influenza viral loads in the lungs were determined as described previously^32,43^. To measure the SARS-CoV-2 viral load, monolayers of Vero TMPRSS2 cells were prepared by seeding 1.425 × 10^6^ cells in 3 mL DMEM and incubated overnight at 37 °C in 5% CO_2_. Homogenized lung samples were diluted tenfold (10−^1^–10−^3^) and then transferred to 6-well plates containing a monolayer of Vero TMPRSS2 cells. The neutralization assay protocol outlined above was followed for the remaining plaque assay. Following incubation for 3 days at 37 °C, 5% CO_2_, visible plaques were counted manually, and the number of PFUs was determined.

### Measurement of gene expression levels using real-time RT-PCR

Fresh lung tissues (right lobes) were collected at necropsy, placed in TRIzol (ZYMO Research, Orange, CA, USA), homogenized using stainless beads by Micro Smash (MS-100/100R; Wakenyaku Co., ltd., Kyoto, Japan), and stored at –80 °C until use. Total RNA was extracted according to the manufacturer’s instruction using the classical chloroform/isopropanol precipitation method, and the RNA concentrations were measured by Nanodrop One spectrophotometer (Thermo Fischer Scientific). The extracted RNA was reverse-transcribed using a High-Capacity cDNA Reverse Transcription Kit (Thermo Fischer Scientific) to prepare cDNA. The synthesized cDNA was subjected to real-time PCR reactions for quantification of gene expression levels of *Csf2*, *Csf3, Il1b, Il6, Il10, Ifng, Tnf*, *Cxcl10*, *Ccl2*, *Icam1* by using a StepOne Real-Time PCR System (Applied Biosystems, Foster City, CA, USA) and QIAGEN Quantinova SYBR green PCR kit (QIAGEN, Hilden Germany). The primers used in this study are shown in Supplemental Table 1. The relative expression levels of target genes were normalized in each sample by those of ribosomal *18S* and non-infected control samples and then calculated as the fold change (2^–∆∆Ct^).

### Histopathology and immunohistochemistry

Lung samples (left lobe) were harvested from non-infected control mice and from infected mice at 3, 5, and 10 dpi and fixed in masked formalin (Japan Tanner, Osaka, Japan) for 7 or 10 days at room temperature. For histopathological analyses, the tissues were paraffin-embedded, sectioned, and stained with H&E. Immunostaining was performed for the detection of viral antigens and inflammatory cell markers (monocytes and neutrophils). Unstained paraffin-embedded lung sections were immersed in Trilogy (Cell Marque, Sierra College Boulevard, Rockline, California) for deparaffinization and antigen retrieval in an antigen retriever (2100 Antigen Retriever; Aptum Biologics, Southampton, UK). To block endogenous peroxidase, sections were placed in 0.3% hydrogen peroxide (Fujifilm Wako Pure Chemical Corp., Tokyo, Japan) in methanol (Sigma Aldrich, St. Louis, MO, USA) for 20 min at room temperature after which they were probed with anti-influenza A, B (1:2000, M149, rabbit polyclonal antibody; TaKaRa Bio inc., Shiga, Japan), anti-nucleocapsid protein of SARS-CoV-2 (1:1000, HL344, rabbit monoclonal antibody; GeneTex, Irvine, CA, USA), anti-F4/80 (1:500, 70076, rabbit monoclonal antibody; Cell Signaling Technology, Danvers, Massachusetts, USA) or anti-Ly-6G (1:500, 87048, rabbit monoclonal antibody; Cell Signaling Technology) antibodies and incubated overnight at 4 °C. Following washing with 0.01 M PBS (Fujifilm Wako Pure Chemical Corp.) three times, sections were incubated with TaKaRa POD conjugate (MK205, anti-rabbit Ig-Fab-peroxidase conjugate; TAKARA Bio. Inc.) for 30 min at room temperature. After washing with 0.01 M PBS, the sections were incubated with TaKaRa DAB substrate (TaKaRa Bio. Inc.) for 5 min at room temperature and then placed in distilled water to stop the reaction. Counterstaining was performed with hematoxylin (Mayer’s Hematoxylin Solution; Fujifilm Wako Pure Chemical Corp.) for 30 min. Finally, the sections were washed with running water, dehydrated with isopropanol. A small amount of a permanent mounting medium (VectaMount Express, Vector Laboratories, Inc., Burlingame, CA, USA) was added and the slides were then covered with a coverslip.

The degree of lung damage was assessed by examining 10 randomly selected fields on H&E-stained sections from each mouse using the established criteria^58–60^ as follows: 0, normal lung; 1, mild destruction of epithelium of the bronchus; 2, mild infiltration of inflammatory cells around the periphery of bronchioles; 3, moderate infiltration of inflammatory cells around the alveolar walls resulting in alveolar thickening; 4, mild alveolar injury accompanied by vascular damage of ≤ 10%; 5, moderate alveolar and vascular injury (11% to approximately 30%); 6, severe alveolar injury with hyaline membrane–associated alveolar haemorhage (31% to approximately 50%); and 7, severe alveolar injury with hyaline membrane-associated alveolar hemorrhage of ≥ 51%. Lung sections from each mouse were assessed and scored individually, and the cumulative total average of 10 areas was reflected as the score for each mouse. The frequencies of macrophages and neutrophils in lung sections were quantified by QuPath bioimage analysis software^61^.

### Statistical analysis

All statistical analyses were performed using GraphPad Prism 10 software. Data are presented as mean with SD. Normality of distribution was checked by Shapiro–Wilk test then data were analysed by Kruskal–Wallis test followed by Dunn’s multiple comparisons test, two-way ANOVA followed by Sidak’s multiple comparison test, and Mann–Whitney test. For multiple comparisons test, each group was compared with every other group. Significant difference was denoted by *p* values < 0.05.

### Supplementary Information

Supplementary Figure S1.

Supplementary Figure S2.

Supplementary Figure S3.

Supplementary Figure S4.

Supplementary Table 1.
