# Supplementary material for: Immunogenicity and protective efficacy of a co-formulated two-in-one inactivated whole virus particle COVID-19/influenza vaccine
Source: Sci Rep. 2024 Feb 20;14:4204. doi: 10.1038/s41598-024-54421-1 (PMC10879490; doi:10.1038/s41598-024-54421-1)
Supplement: Supplementary file 1 — Supplementary Figure S1. [file 41598_2024_54421_MOESM1_ESM.pdf]

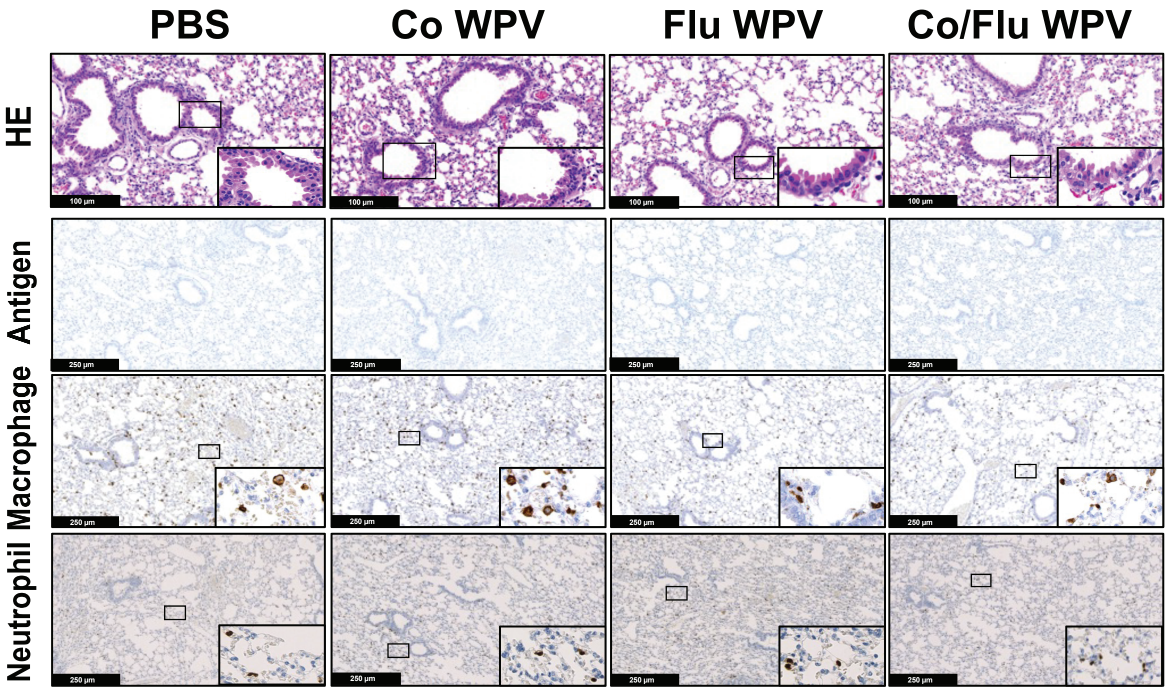


**Supplemental Figure S1.**

**Histopathology and immunohistochemistry of lung sections from non-infected control mice.**

Female BALB/c mice were immunized subcutaneously with Co WPV, Flu WPV, Co/Flu WPV or PBS as the control group, and lung tissues were harvested on day 19 post-vaccination (n = 3/group). For histopathological analysis, lung sections were stained with H&E, and immunohistochemistry was performed to detect SARS-CoV-2 nucleocapsid and macrophage (F4/80) and neutrophil (Ly-6G) markers. Each lung section is a representative of 3 lung sections analysed.
