# Supplementary material for: Immunogenicity and protective efficacy of a co-formulated two-in-one inactivated whole virus particle COVID-19/influenza vaccine
Source: Sci Rep. 2024 Feb 20;14:4204. doi: 10.1038/s41598-024-54421-1 (PMC10879490; doi:10.1038/s41598-024-54421-1)
Supplement: Supplementary file 2 — Supplementary Figure S2. [file 41598_2024_54421_MOESM2_ESM.pdf]

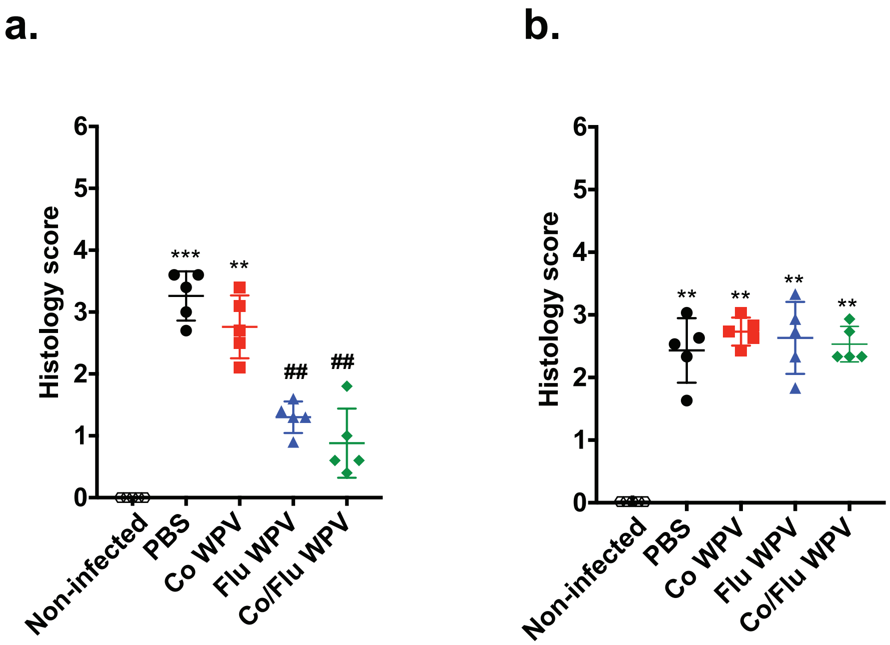

Supplemental Figure S2.
Histopathology scores after influenza virus or SARS-CoV-2 infection
Female BALB/c mice were immunized subcutaneously with Co WPV, qFlu WPV, Co/qFlu WPV, or PBS as the control group (n = 5/group). At 22 days post-vaccination, animals were infected with 3,000 PFU of A/California (H1N1) or 10^5 PFU of SARS-CoV-2 MA-P10. The degree of lung pathology after influenza virus (a) or SARS-CoV-2 challenge (b) was assessed on H&E-stained lung sections using the criteria described in the methods section. Statistical analysis was performed using Kruskal-Wallis test with Dunn's multiple comparisons tests. The * and # symbols indicate statistical significance between the non-infected group and infected groups and between unvaccinated (PBS) and vaccinated groups, respectively. ***p* < 0. 01, ****p* < 0. 001, and ##*p* < 0.01
