# Supplementary material for: Immunogenicity and protective efficacy of a co-formulated two-in-one inactivated whole virus particle COVID-19/influenza vaccine
Source: Sci Rep. 2024 Feb 20;14:4204. doi: 10.1038/s41598-024-54421-1 (PMC10879490; doi:10.1038/s41598-024-54421-1)
Supplement: Supplementary file 3 — Supplementary Figure S3. [file 41598_2024_54421_MOESM3_ESM.pdf]

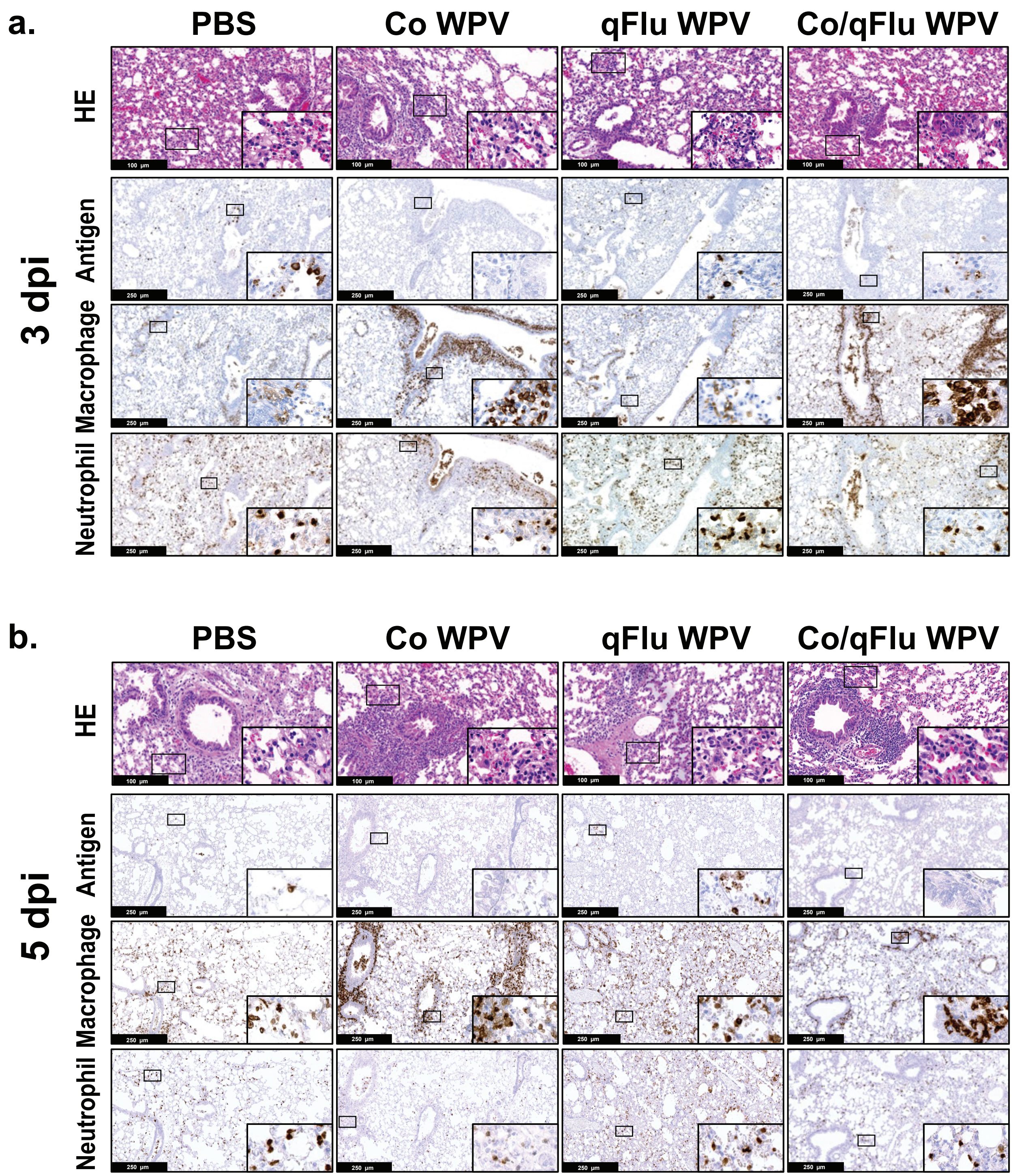


**Supplemental Figure S3.**

**Histopathology and immunohistochemistry of lung sections at 3 and 5 dpi.**

Female BALB/c mice were immunized subcutaneously with Co WPV, qFlu WPV, Co/qFlu WPV, or PBS as the control group (n = 5/group). At 22 days post-vaccination, animals were infected with $10^5$ PFU of SARS-CoV-2 MA-P10. Lung tissues from mice infected with SARS-CoV-2 MA-P10 were harvested at 3 and 5 dpi for histopathology using H&E staining and immunostaining of SARS-CoV-2 nucleocapsid, macrophage (F4/80), and neutrophil (Ly-6G) markers. Each lung section is a representative of 5 lung sections analysed.
