# Supplementary material for: Immunogenicity and protective efficacy of a co-formulated two-in-one inactivated whole virus particle COVID-19/influenza vaccine
Source: Sci Rep. 2024 Feb 20;14:4204. doi: 10.1038/s41598-024-54421-1 (PMC10879490; doi:10.1038/s41598-024-54421-1)
Supplement: Supplementary file 4 — Supplementary Figure S4. [file 41598_2024_54421_MOESM4_ESM.pdf]

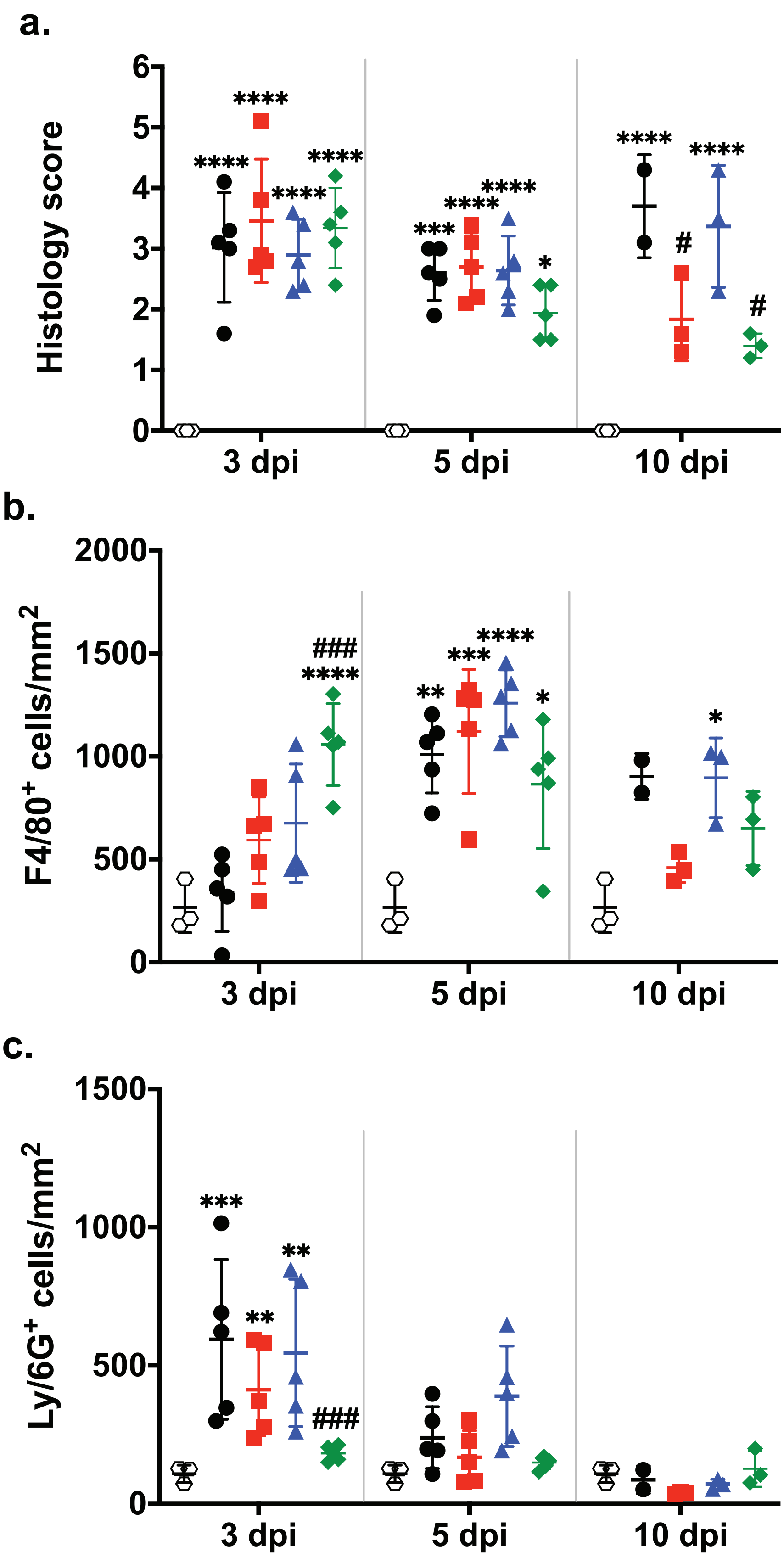

Supplemental Figure S4.

Histopathology scores and frequency of macrophages and neutrophils after SARS-CoV-2 infection. Female BALB/c mice were immunized subcutaneously with Co WPV, qFlu WPV, Co/qFlu WPV, and PBS as the control group (n = 13/group). At 22 days post-vaccination, animals were infected with 10^5 PFU of SARS-CoV-2 MA-P10. The degree of lung pathology was assessed on H&E-stained lung sections using the criteria described in the methods section (a). Macrophages (F4/80+) and neutrophils (Ly-6G+) were quantified by QuPath software (b and c). Mean and SD are shown. Statistical analysis was performed using Kruskal-Wallis test with Dunn's multiple comparisons tests. The * and # symbols indicate statistical significance between the non-infected group and infected groups and between unvaccinated (PBS) and vaccinated groups, respectively. *$p < 0.05$, **$p < 0.01$, and #$p < 0.05$, ##$p < 0.01$.
