# Supplementary material for: Immunogenicity and protective efficacy of a co-formulated two-in-one inactivated whole virus particle COVID-19/influenza vaccine
Source: Sci Rep. 2024 Feb 20;14:4204. doi: 10.1038/s41598-024-54421-1 (PMC10879490; doi:10.1038/s41598-024-54421-1)
Supplement: Supplementary file 5 — Supplementary Table 1. [file 41598_2024_54421_MOESM5_ESM.pdf]

Supplemental Table 1: Primers used in this study

Gene name	Primer sequence	
Csf2 [1]	Forward	CGGCCGGGGAAGCATGTAGA
	Reverse	GCTTGTGTTTCACAGTCCGT
Csf3 [1]	Forward	CATGAAGCTAATGGCCTGC
	Reverse	CTGACAGTGACCAGGGGAAC
Il1b [1]	Forward	TGCCACCTTTTGACAGTGATG
	Reverse	CAAAGGTTTGGAAGCAGCCC
Il10 [1]	Forward	CCAGCTGGACAACATACTGCTA
	Reverse	GAGAAATCGATGACAGCGCC
Il6	Forward	TACCCCAATTTCCAATGCTCTCC
	Reverse	GGATGGTCTTGGTCCTTAGCCA
Ifng [1]	Forward	AGGAACTGGCAAAAGGATGGT
	Reverse	CTGGTGGACCACTCGGATG
Tnf [1]	Forward	AGGCACTCCCCCAAAAGATG
	Reverse	CTTGGTGGTTTGCTACGACG
Cxcl10 [2]	Forward	GCCGTCATTTTCTGCCTCAT
	Reverse	GCTTCCCTATGGCCCTCATT
Ccl2	Forward	TAACGCCCCACTCACCTGCT
	Reverse	TCCTTCTTGGGGTCAGCACA
Icam [3]	Forward	CCGCAGGTCCAATTCACCACT
	Reverse	TCCAGCCGAGGACCATACAG
18S [4]	Forward	GCCGCTAGAGGTGAAATTCTTG
	Reverse	CTTTCGCTCTGGTCCGTCTT

1. Zhang, Y. *et al.* SARS-CoV-2 Rapidly Adapts in Aged BALB/c Mice and Induces Typical Pneumonia. *J Virol* **95**, (2021).

2. Yamaguchi, T. *et al.* ACE2-like carboxypeptidase B38-CAP protects from SARS-CoV-2-induced lung injury. *Nat Commun* **12**, (2021).

3. Ye, Z. *et al.* Increased CYP4B1 mRNA is associated with the inhibition of dextran sulfate sodium-induced colitis by caffeic acid in mice. *Exp Biol Med* **234**, 606–616 (2009).

4. Farina, N. H. *et al.* A role for RNA post-transcriptional regulation in satellite cell activation. *Skelet Muscle* **2**, (2012).
